# *In Vivo* Mesoscopic Voltage-Sensitive Dye Imaging of Brain Activation

**DOI:** 10.1038/srep25269

**Published:** 2016-04-29

**Authors:** Qinggong Tang, Vassiliy Tsytsarev, Aaron Frank, Yalun Wu, Chao-wei Chen, Reha S. Erzurumlu, Yu Chen

**Affiliations:** 1 Fischell Department of Bioengineering, University of Maryland, College Park, MD 20742 USA; 2 Department of Anatomy and Neurobiology, University of Maryland School of Medicine, Baltimore, MD 21201 USA; 3 Department of Electrical and Computer Engineering, University of Maryland, College Park, MD 20742 USA

## Abstract

Functional mapping of brain activity is important in elucidating how neural networks operate in the living brain. The whisker sensory system of rodents is an excellent model to study peripherally evoked neural activity in the central nervous system. Each facial whisker is represented by discrete modules of neurons all along the pathway leading to the neocortex. These modules are called “barrels” in layer 4 of the primary somatosensory cortex. Their location (approximately 300–500 μm below cortical surface) allows for convenient imaging of whisker-evoked neural activity *in vivo*. Fluorescence laminar optical tomography (FLOT) provides depth-resolved fluorescence molecular information with an imaging depth of a few millimeters. Angled illumination and detection configurations can improve both resolution and penetration depth. We applied angled FLOT (aFLOT) to record 3D neural activities evoked in the whisker system of mice by deflection of a single whisker *in vivo*. A 100 μm capillary and a pair of microelectrodes were inserted to the mouse brain to test the capability of the imaging system. The results show that it is possible to obtain 3D functional maps of the sensory periphery in the brain. This approach can be broadly applicable to functional imaging of other brain structures.

Visualization of evoked and spontaneous neuronal activity *in vivo* is of great importance for understanding brain functions. Sensory pathways in the mammalian brain have been particular targets of imaging studies, because neural networks can be evaluated following specific stimulation of the sense organs, which translate, and transmit physical energy to the brain. One such sensory pathway, highly amenable to experimental manipulations, is the rodent whisker-barrel system. Whisker-specific sensory afferents and their postsynaptic partners form discrete physical representations in the brainstem, thalamus and neocortex^1^. Thus, point-to-point and topographic representation of the activation of the sensory periphery in the brain can be evaluated with electrophysiological and imaging approaches.

Voltage-sensitive dye imaging (VSDi) has been quite useful in imaging activities of the neural networks in the brain^2^. VSDi is based on fluorophore molecules that bind to the neural membrane and convert changes in transmembrane voltage into the fluorescence of the emitted light^2^. VSDi creates an opportunity to monitor neural activity *in vivo* with relatively high spatial and temporal resolution (up to microseconds). However, VSDi, being a variety of the wide field optical imaging technique, cannot allow visualization of neural activity in three dimensions.

Mesoscopic imaging has been developed in recent years, including mesoscopic epifluorescence tomography^3^,^4^, and fluorescence laminar optical tomography (FLOT)^5^,^6^,^7^. An array detector with different separations from the light source is used in FLOT, which enables simultaneous detection of scattered light from different depths. FLOT can achieve an axial resolution of ~100–200 microns with several millimeter imaging depth^8^, and has been extended to time-resolved imaging in the perfused rat heart with voltage-sensitive dyes (VSDs) to obtain 3-dimensional (3D) propagation of electrical waves^5^. Recent studies indicate that angled illumination and detection configurations can improve both resolution and depth sensitivity^9^.

In this paper, combining aFLOT with VSDs, we present 3D neural activities evoked in the whisker barrel cortex of mice by deflection of a single whisker *in vivo* with 5 ms temporal resolution (e.g., an effective frame rate of 200 Hz). Further, our results lend support to the hypothesis that a 3D dynamic activity patterns, represent neural network processing in different parts of the cerebral cortex.

## Results

### 3D characterization of the aFLOT system

**Figure 1:**
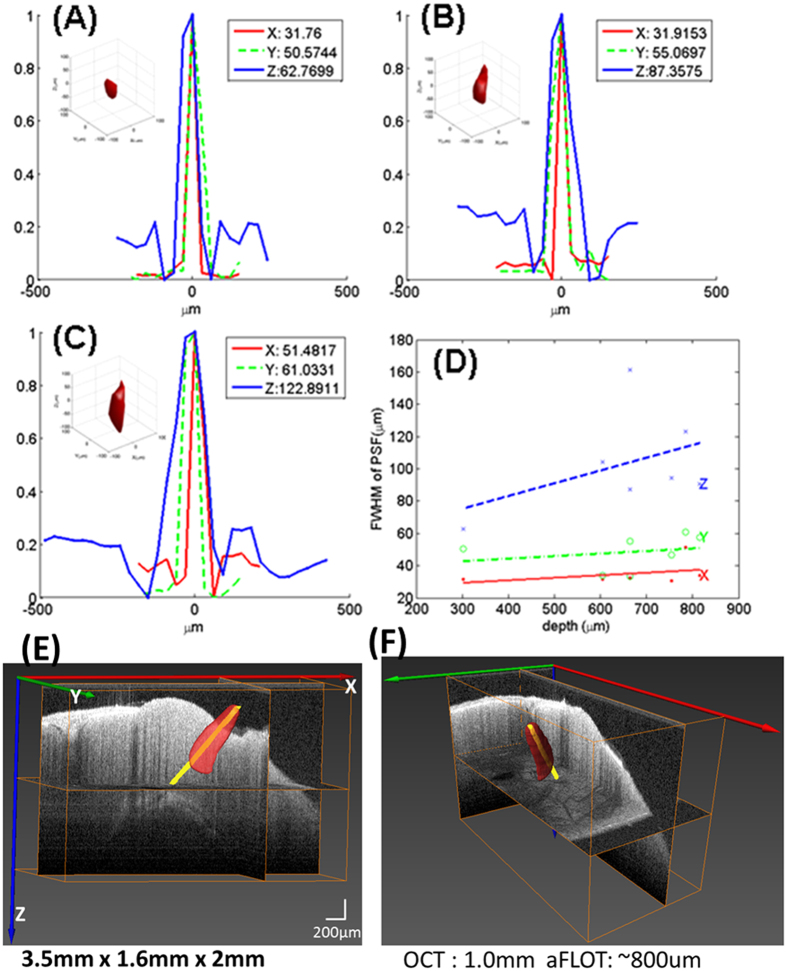
3D PSFs of the aFLOT system at 302 μm (**A**), 664 μm (**B**), and 785 μm (**C**). Insets show the isosurface of PSFs with μ_s_′ = 0.5/mm. Legends report FWHM in μm in x, y, and z directions. (**D**) FWHM versus depths. •, ○, and × represent respectively the FWHM in x, y, and z directions of a single PSF at the corresponding depth. (**E**,**F**) Reconstructed 3D aFLOT fluorescence images of 100 μm glass capillary tube superimposed with OCT data.

We used quantum-dot-embedded hydrogel to characterize the point spread function (PSF) of our aFLOT system. Recipe of this phantom was 35 μL QD705 (QD705, Invitrogen), 1.5 mL poly(ethylene glycol) diacrylate (PEGDA), 3.5 mL water, 1 mL ammonium persulfate (APS) (0.2 M), and 1 mL N,N,N′,N′-tetramethylethylenediamine (TEMED) (0.2 M). The mixture was vortexed and immediately poured into well plate as it crosslinked within 30 s. The measured reduced scattering coefficient (μ_s_′) of the hydrogel was 0.5/mm. For aFLOT imaging, the emission filter was first removed and the field of view (FOV) was scanned to get the reflectance images with the step size 46 μm. Only one image was needed at each scanning position for reflectance mode. To record the fluorescence images for quantum dots, we intended to obtain 3D reconstruction of the static quantum dots (i.e., the fluorescence signal is not time-dependent), thus only one image at each scanning position was acquired after adding the emission filter^10^. Figure 1(A–C) show 3 PSFs of the quantum dots at depths of 302, 664, and 785 μm, respectively. Full width at half maximum (FWHM) in x, y, and z directions mildly increases toward deeper depths (Fig. 1(D)). Pixelation of PSF was observed. This however suggests that true PSF would be slightly smaller than the values reported here. We observed a trend of increase for axial PSF with depth, reaching ~120 μm at 800 μm depth. Furthermore, we also observed in general, PSF in y direction is larger than x direction, which is due to the asymmetric illumination between x and y direction.

### 3D reconstruction of 100 μm glass capillary in mouse brain

Since the optical property of the living mouse brain is complicated, it is difficult to make a phantom to simulate this sophisticated biological machinery. Therefore we carried out an experiment using a 100 μm glass tube to verify the performance of the aFLOT system in the living mouse brain. The 100 μm glass capillary tube (ID: 0.1 mm, OD: 0.17 mm, Vitrocom Inc.) was filled with VSD and fixed on a 3D manipulator. The capillary was then inserted into a mouse brain, which was handled through the animal preparation protocol except dye loading. In this experiment, we first used OCT to scan the glass capillary tube in the brain. Then the mouse brain with the capillary tube was used for the aFLOT experiment. For aFLOT imaging, the emission filter was first removed and the field of view (FOV) was scanned to get the reflectance images with the step size 46 μm. Only one image was needed at each scanning position for reflectance mode. To record the fluorescence images for capillary, we intended to obtain 3D reconstruction of the static capillary structure (i.e., the fluorescence signal is not time-dependent), thus only one image at each scanning position was acquired after adding the emission filter^10^. The reflectance images of the FOV ware used to reconstruct the surface feature, which was used to align the 3D OCT data with the 3D aFLOT fluorescence data. Figure 1(E,F) shows different perspectives of the reconstructed 3D aFLOT fluorescence images superposed with the optical coherence tomography (OCT) data. OCT can provide information about the brain structure and depth. The yellow object in the 3D OCT image is the capillary recorded by OCT and the red one is the aFLOT reconstructed capillary tube. OCT can image around 1 mm deep in the living mouse brain while aFLOT around 800 μm in depth, without major image distortion; barrels are about 300–500 μm deep from the pial surface in adult mice.

### Time-resolved 3D reconstruction of electrode stimulation in the mouse brain

**Figure 2:**
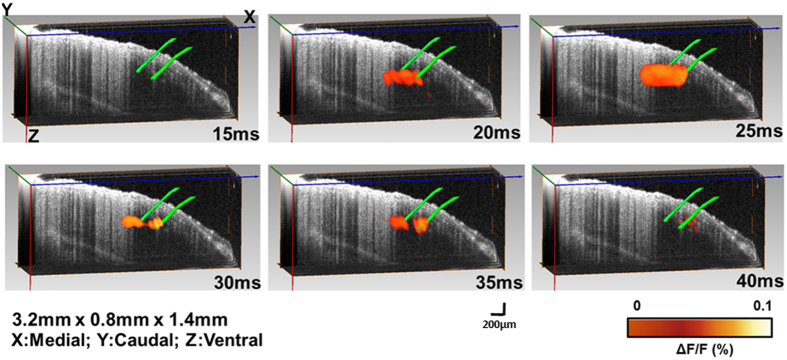
3D changes in fluorescence (ΔF/F(%), ordinate) in response to electrical stimulation reconstructed by aFLOT system superimposed with OCT data. Time period after stimulation is indicated at the bottom right corner of each image.

We verified the overall process for 3D functional imaging using a pair of electrodes. The electrodes were first fixed on a 3D manipulator, and then the electrodes were inserted into the mouse brain. Similar to the capillary tube experiment, we first used OCT to get the 3D electrode image in the brain and then used aFLOT to image the mouse brain with the electrodes. For aFLOT imaging, the emission filter was first removed and the FOV was scanned to get the reflectance images with the step size of 46 μm, with only one image at each scanning position used for the reflectance mode. Then the emission filter was applied, and based on the data acquisition protocol for time-resolved aFLOT imaging (described in the Methods section), the fluorescence images were recorded. The reflectance image of the FOV was used to reconstruct the surface features, which were used to align the 3D OCT data with the 3D aFLOT fluorescence data. Figure 2 shows the electrodes reconstructed with 3D aFLOT in the fluorescence images superposed with OCT data. OCT shows that the electrode tips are at around 440 μm depth from the cortical surface. The two green rods in the 3D OCT image are the electrodes recorded by OCT and the orange ones are the aFLOT-reconstructed changes in fluorescence (ΔF/F(%), ordinate) in response to electrical stimulation at different time points. The signal appeared about 15 ms after the stimulus onset, and reached peak at 25 ms after the stimulus onset. At time 30 ms and 35 ms we can clearly see a pair of signals surrounding the two electrode tips, which indicate that aFLOT system and imaging protocol can be used to record 3D functional images. The activated signals disappeared gradually, after 40 ms.

### Time-resolved 3D reconstruction of cortical neural activity evoked by single whisker deflection

**Figure 3:**
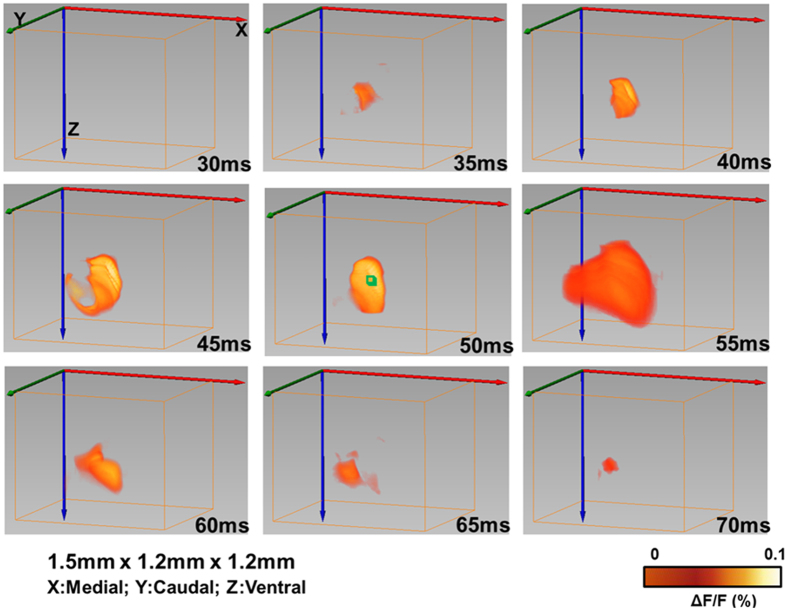
3D changes in fluorescece (ΔF/F(%), ordinate) in response to the C2 whisker stimulation reconstructed by the aFLOT system. Time period after stimulation is indicated at the bottom right corner of each image.

**Figure 4:**
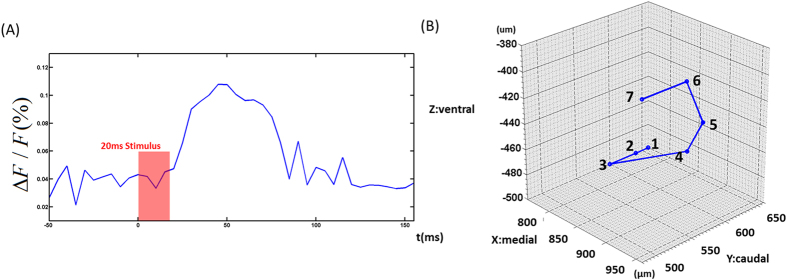
(**A**) Change in fluorescence (ΔF/F(%), ordinate) in response to the C2 whisker stimulation. Fluorescence signal was calculated from the ROI (green cubic box: 3 × 3 × 3 pixels) shown in Fig. 3 at 50 ms post-stimulation. (**B**) Coordinates of the strongest change in fluorescence (ΔF/F(%), ordinate) in response to the C2 whisker stimulation. 1:30 ms and 35 ms (760 μm, 660 μm, −500 μm); 2:40 ms (760 μm, 640 μm, −500 μm); 3:45 ms (760 μm, 600 μm, −500 μm); 4:50 ms (940 μm, 560 μm, −440 μm); 5:55 ms (900 μm, 620 μm, −440 μm); 6:60 ms and 65 ms (960 μm, 540 μm, −380 μm); 7:70 ms (960 μm, 480 μm, −380 μm).

After verifying the imaging ability of the aFLOT system, for both structure and function, aFLOT was used to record 3D neural activities evoked in the barrel cortex by deflection of a single whisker *in vivo*. The C2 whisker was used for 20 ms stimulation. Figure 3 shows the 3D aFLOT reconstructed changes in fluorescence (ΔF/F(%), ordinate) in response to C2 whisker stimulation at different time points. The signal appeared about 30 ms after the stimulus onset, and reached its peak at 50 ms after the stimulus onset. After 70 ms, the activated signals disappeared gradually. The time course corresponds well with the previously published data^11^. Figure 4(A) shows the change in fluorescence (ΔF/F(%), ordinate) in response to the C2 whisker stimulation. Fluorescence signal was calculated from the region of interest (ROI, green cubic box: 3 × 3 × 3 pixels) shown in Fig. 3 at 50 ms post-stimulation. Figure 4(B) shows coordinates of the largest changes in fluorescence (ΔF/F(%), ordinate) in response to the C2 whisker stimulation. Figure 4(B) shows that the signal center was at around 500 μm below the cortical surface at 30–45 ms and then moved to around 440 μm below the cortical surface at 50 and 55 ms. Finally, the center of the signal moved to around 380 μm below the cortical surface.

**Figure 5:**
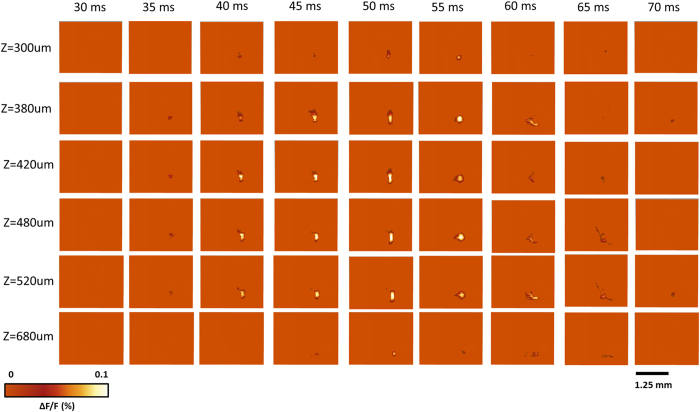
Changes in fluorescence (ΔF/F(%), ordinate) in response to the C2 whisker stimulation reconstructed by the aFLOT system in different depths. Time period after stimulation is indicated at the top of the whole image. The left column shows the depth information.

To better visualize the depth-resolved neural activity, changes in fluorescence (ΔF/F(%), ordinate) in response to the C2 whisker stimulation were reconstructed by aFLOT in different depths (XY cross-sections) as shown in Fig. 5. The changes in fluorescence first took place in the range 380–520 μm at about 35–40 ms, then the response became stronger in both deeper (680 μm) and shallower region (300 μm and 380 μm) at around 50 ms and 55 ms. Although the signal response trend existed at depth of 300 μm and 680 μm, the average response intensity was weaker compared to the other depths, which may be caused by the difference of neuronal density at different depths or in layers. Also, we observed that the neural responses in the 420–520 μm range lasted longer than in other depths.

**Figure 6:**
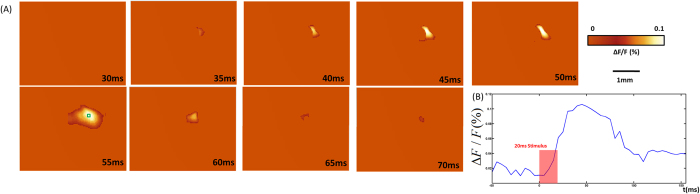
(**A**) Changes in fluorescence (ΔF/F(%), ordinate) in response to C2 whisker stimulation reconstructed by aFLOT system in XZ cross-section. Time period after stimulation is indicated at the bottom right corner of each image. (**B**) Change in fluorescence (ΔF/F(%), ordinate) in response to the C2 whisker stimulation. Fluorescence signal was calculated from the ROI (green square: 3 × 3 pixels) shown in (**A**) at 55 ms post-stimulation.

Figure 6(A) shows changes in the fluorescence (ΔF/F(%), ordinate) in response to C2 whisker stimulation reconstructed by aFLOT in the XZ cross-section. Figure 6(B) shows the change in fluorescence (ΔF/F(%), ordinate) intensity in response to C2 whisker stimulation, in the middle XZ cross-section. The fluorescence signal was calculated from the ROI (green square: 3 × 3 pixels) shown in Fig. 6(A) at 50 ms post-stimulation. Compared to neural responses in XY plane, XZ cross-section provides us with a new perspective to investigate the neural activities at different time points.

## Discussion

Development of VSDi and other 3D imaging techniques enable examination of spatiotemporal patterns of neuronal activities in the brain. We have demonstrated that it is possible to detect the 3D neural activities by deflection of a single whisker using non-contact optical measurements of the mouse brain *in vivo*.

Time-resolved FLOT has been used in the perfused rat heart with VSDs to obtain 3-dimensional (3D) propagation of electrical waves^5^. In this study, we applied time-resolved FLOT but with angled illumination configuration and CCD camera as detectors which promise to improve both resolution and penetration depth^9^. We have demonstrated the 3D imaging capabilities of our aFLOT system using a quantum-dot-embedded hydrogel. Since the resolution and penetration depth depend on the optical properties of sample, an ideal sample to test the feasibility of the aFLOT system would be a living mouse brain. As illustrated in Fig. 1 (E,F), the reconstructed 100 μm glass capillary tube by aFLOT was compared with the image acquired by OCT. The accuracy and resolution of aFLOT degrade with depth. We can observe the reconstruction distortion was within ±120 μm up to 800 μm depth, which definitely covered the Layer IV in mouse somatosensory cortex. Performance of our aFLOT system is quite remarkable, despite limitations, and future work will incorporate the newer NIR and red-shifted VSDs to achieve significantly improved penetration of light into the mouse brain.

In addition to the 3D static system performance evaluation, we observed a rapid response to direct electrical stimulation through the use of a bipolar electrode in the living mouse brain. A study of the intracortical microsimulation provided insight into the spatial extent of the excited neural tissue and its relationship to the exciting current^12^. The threshold gradient across neurons 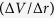 is constant and is related to the applied current as: 
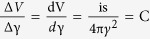
 , where 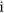 is the applied current, 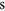 is the specific resistivity of the tissue, and 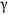 is the distance from the current source to the neuron^12^. Thus, the relationship between a particular neuron’s threshold current and the neuron’s distance from the tip of the stimulating electrode can be expressed as: *i* = K*γ*^2^, in which *K* = 4Cπ/s. For mammalian brain tissue, the 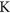 has a value of ~1200 μA/mm^2^ 12. Thus, if the stimulation current is 100 μA, more than half of the neurons will be excited in the sphere with a radius of 288 μm from the tip of the electrode^12^,^13^. These computations do not take into account the frequency of the stimulation, as well as 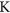 having the possibility of being slightly different in different brain tissues. They also do not take into account the influence of pulse duration on the neural excitation and the level of the threshold used for analysis. Nevertheless the computation is generally correct as can be seen in Fig. 2 (the size of the changes in fluorescence at 25 ms). Since bipolar electrode consists of two metal needles, isolated at all longevity except the tips, there are two metal tips in the brain and both stimulated the brain tissues simultaneously. The size of the fluorescence changes at 25 ms was around 1000 μm in length through the direction of the two tips of the electrodes and around 450 μm in z direction. Calculated 288 μm was compared with the distance of fluorescence border to the needle tip (~250 μm in radius). By comparing the spatial extent of the excited neural tissue with the calculation data mentioned above, we were able to evaluate the likely accuracy of our 3D imaging performance.

The voltage-sensitive dye spread along the mediolateral and dorsoventral axes of the neocortex. It was shown that the voltage-sensitive dye RH-1691 loading extended across all cortical layers, but the dye concentration in deep layers (V–VI) is lower^14^,^15^. Neurons within layer IV directly respond for whisker movement, in other words, many neurons of this layer will fire when the whisker is mechanically stimulated. The strongest inputs from the thalamus carrying information from a facial whisker to the barrels of layer IV, but the inputs from the thalamus to the layers III, V and VI also exist^15^. Excitatory signals from the thalamus reach different layers of the barrel cortex at different times. From Fig. 5 we can observe the neural responses in the range 420–520 μm which represents the layer IV last longer and have stronger than other depths (shallower and deeper depths). The weaker response in deeper layers (V–VI) may originate from both less neurons corresponding to whisker movement and also less dye concentration. The intensity of the responses at different depths can be more intuitionally visualized in XZ cross-section in Fig. 6(A) at 55 ms.

Also we can see that in Fig. 3, the VSDi activity patterns in the cortical area evoked by stimulating a single whisker is larger than the corresponding barrel^16^,^17^. The larger response size than barrel in our experiment may also partially caused by the resolution of our reconstruction.

An important question in sensory information processing is how sensory information is represented and processed in the neocortex. The manner by which sensory-evoked neural activity propagates across cortical layers remains unclear^18^ . During the last few decades, propagating waves have been observed during different types of cortical processing as revealed by imaging or electrophysiological methods^19^. The spiral-like waves, as well as more complex activity patterns have been visualized by VSDi *in vivo* in the turtle visual cortex^20^.

In our experiment, by localizing the coordinates of largest fluorescence signals at different time point, we were able to show the progress of sensory activity in the neocortex. As shown in Fig. 4(B), we can see the movement of the activated area in both horizontal and vertical directions. The movements of signal centers at different time points are particular kind of propagating waves that rotate around a center point. Although the resolution of our aFLOT system is not high enough to demonstrate the propagating wave, for the first time we demonstrated the propagating trajectory in 3D manner and our data agrees with the electrophysiological data recently obtained using multi-electrode arrays^18^ . Future work will focus on further improving the resolution of our aFLOT system.

## Methods

All experiments were performed in accordance with the National Institutes of Health Guide for the Care and Use of Laboratory Animals (NIH Publication No. 80-23) and a protocol approved by the Institutional Animal Care and Use Committee of the University of Maryland, Baltimore and College Park campuses.

### Animal preparation

Experiments were performed on five B6 mice at 2–3 months of age. Animals were anesthetized with urethane (1.15 g/kg body weight). Their heads were shaved before placing in a stereotaxic frame (Stoelting Ltd)^14^. A cranial window (about 3 × 3 mm) was made over the left parietal cortex, and the surface of the dura matter was cleaned with hemostatic sponge dipped in artificial cerebrospinal fluid (ACSF). Voltage-sensitive dye RH-1691 (Optical Imaging Ltd; 1.0 mg/ml in ACSF) was applied to the exposed area for 45 min^14^. After staining, the cortex was washed with dye-free ACSF for 15 min and the cortical surface was covered with high-density silicone oil and then sealed with a 0.1-mm-thick cover glass. A glass pipe (1.0 mm in diameter) fitted onto an XYZ manipulator was aimed at the facial C2 whisker. An air-puff stimulus of a 20 ms duration was applied through a Picospritzer pressure valve connected to the glass pipette^14^. In the electrode stimulation experiments, a bipolar tungsten electrode (containing two electrodes) (FHC Ltd, Bowdoin, US) was inserted into the brain by using a micromanipulator (Narishige, Japan). Electrical currents were applied through both electrodes. The stimulation system was coupled to the computer of the imaging system.

### Experimental setup

**Figure 7:**
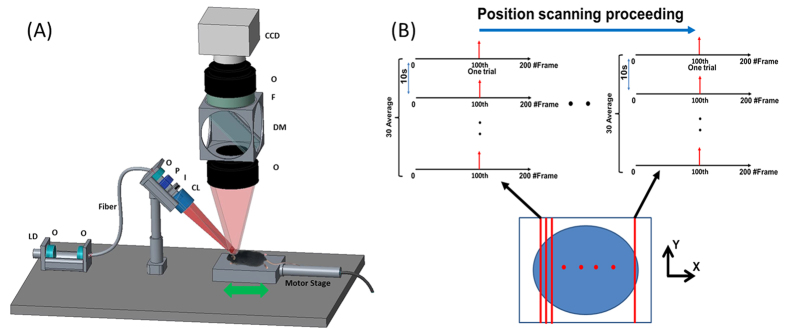
(**A**) Schematic of the aFLOT system. LD: laser diode; O: objective lens; P: polarizer; I: iris; CL: cylindrical lens; F: filter; DM: dichroic mirror. CCD is for capturing both laminar fluorescence and reflective light distribution (by removing F). The illumination arm is arranged at 45° in air. (**B**) Image acquisition protocol.

Figure 7(A) shows the schematic diagram of the aFLOT system. A 637 nm laser diode was utilized as the light source; the light was collimated and coupled into a single-mode fiber to shape the light beam. Light coming out from the fiber was first collimated by an objective lens and passed through a polarizer, which was used to reject the specular reflection from the surface. The collimated light was expanded into line-field illumination using a cylindrical lens with a full line-width at the half maximum of 26 μm at the focal plane. An iris was used to control the length of the line illumination. The emitted fluorescent light was then collected back through the objective lens, dichroic mirror (650 nm, single edge dichroic beam splitter; FF650-DiO1-50 × 70 mm; Andover Corporation), emission filter (695 nm, 695FG07-50, Andover Corporation), and finally imaged to a high-speed CCD camera (MiCAM02-HR, SciMedia, Ltd). The laser diode and emission filter used for quantum dots were 649 nm and 705 nm respectively. The illumination angle was set at 45°, rendering ~30° transmission angle in tissue (n~1.33). The CCD camera was placed vertically to record both fluorescence and reflectance images by changing the filter. The reduced scattering coefficient (μ_s_′) of the sample was determined from the reflectance data using oblique-incidence spectroscopy^21^. A motor stage was used to translate the sample laterally in scanning direction (perpendicular to the line illumination direction). OCT can provide depth-resolved cross-sectional images of tissue microstructures with micrometer resolution^22^,^23^. In the glass capillary and electrode experiments, the living mouse brain was first scanned with the OCT system to get 3D images, which provided the structure information of the mouse brain. The 3D images can also indicate the shape and tip location of the glass capillary and electrodes which served as the reference for the following results from aFLOT experiments in the same mouse brain. The OCT system used here utilized a wavelength-swept laser source (Thorlabs, Inc.), which generated a broadband spectrum of 100 nm FWHM centered at 1310 nm, and has been described previously^10^,^24^.

### Stimuli and data acquisition

For 3D reconstruction of 100 μm glass capillary in the mouse brain, since the fluorescence signal is static (i.e., no time-dependent information), only one image at each scanning position was needed^7^. The CCD pixel size was 23 um. In order to scan long enough (~2.7 mm) area in scanning direction and save the overall data recording time, 46 μm was chosen as the step size. Typical scanning protocol was 60 steps with a step size of 46 μm, which equaled to ~2.7 mm total movement in scanning direction. The raw measurement for glass capillary imaging had the format of XYS_j_ (where X and Y represent the X and Y dimensions of the 2D image acquired by CCD, S represented the different scanning position, X = 184; Y = 128; j = 1, …, 60). Since the optical properties were different in air and brain, in order to compare the theoretical model with experimental data as precise as possible, brain surface must be found. To obtain the surface tomography of the mouse brain, the emission filter was removed and the same FOV was scanned to get the reflectance images with the same scanning step size. Without emission filter, most the collected light was from the reflection of the illumination light at the brain surface, which would then serve as an indicator of the location of brain surface. The raw measurement of reflectance mode had exactly the same format of XYS_j_ (X = 184; Y = 128; j = 1, …, 60). The image acquisition protocol for time-resolved aFLOT was shown in Fig. 7(B). The illumination line was first focused on the border of the desired field of view (FOV). At each scanning position, an experimental session was finished which consisted of 30 trials, with 200 frames per trial and 5 ms/frame. In each trial, the stimulus (the train of square electrical pulses, 100 μA, 0.3 ms, 300 Hz, 10 ms train duration) or 20 ms whisker deflection was presented at the 100^th^ frame (one stimulus per trial). The images from these 30 trials were averaged to obtain the response XYT_i_S_0_ (where X = 184; Y = 128; i = 1, …, 200). Next, the motor stage moved 46 μm in scanning direction to another illumination/collection area and another experimental session was performed to obtain the next dataset XYT_i_S_1_ (where X = 184; Y = 128; i = 1, …, 200). This process was repeated until finishing the entire FOV scanning. The raw measurement had the format of XYT_i_S_j_ (where X = 184; Y = 128; i = 1, …, 200; j = 1, …, 60) for electrodes and whisker imaging. Finally, because of defocus effect, we set the focus plane slightly below the dural surface.

### Data reconstruction and analysis

The raw measurements of the reflectance data were simply stacked, according to the geometrical relationship between the illumination plane and detection FOV, which was similar to the unprocessed stacked raw image in selective-plane illumination microscopy^25^. Additionally, for the experiment of glass capillary and electrodes, the stacked reflectance data were used to co-register with the OCT data based on the shape / structure / insertion feature of the glass capillary and electrodes. To reconstruct the 3D fluorescence distribution, the first step was to match the step size and the pixel size; interpolation was then done only in the scanning dimension changing the raw dataset from XYS_j_ (X = 184; Y = 128; j = 1, …, 60) to XYS_j_ (where X = 184; Y = 128; j = 1, …, 120) for glass capillary imaging using a custom MATLAB algorithm. To reconstruct the images, we assumed first-order Born approximation to obtain linearity between the measurement 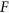 and the fluorophore distribution 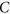, which is the FOV_XZ_ to be reconstructed. This linear relationship is written as *F* = *JC* ,where 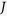 is the weight or sensitivity matrix. To constitute 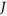, photon distribution was first generated by Monte-Carlo simulation (g = 0.9, n = 1.33, μ_a_ = 0.01/mm, μ_s_′ = 0.82/mm)^26^. Then we applied the reciprocity principle.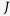 was later decomposed by singular value decomposition (SVD)^27^. Lastly, least square fitting and Tikhonov regularization^9^ were used to solve the underdetermined system^9^. The regularization parameter α = 0.0016 was determined by L-curve criterion^28^. 100 source-detector pairs and 100 scanning positions were chosen to constitute 10,000 measurement modes. Each reconstructed FOV_XZ_ was 100 × 100 pixels with a pixel size of ~23 μm. Weight matrix 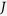 is therefore of size 10000 × 10000. FOV_XYZ_ was constituted by superimposing individual FOV_XZ_ in Y direction. Since the reflectance images and fluorescence images have exactly the same format, the reflectance images, which can indicate the location of the brain surface can be used to select the ROI for fluorescence images to perform reconstruction using our custom MATLAB algorithm. For the time-resolved aFLOT experiment in electrodes and whisker stimulation, since fluorophore intensity changes were used to indicate the activated area in VSDi, the original fluorophore intensity needed to be calculated first. For each XYT dataset at each scanning position, the final ten pre-stimulus frames (90–99^th^ frame) were averaged as the baseline image. The baseline image was then subtracted from each subsequent frame to obtain changes in fluorescence signals. The next step was to match the step size and pixel size; interpolation was then done only in the scanning dimension changing the raw dataset to XYT_i_S_j_ (where X = 184; Y = 128; i = 1, …, 200; j = 1, …, 120) for electrodes and whisker imaging using a custom MATLAB algorithm. The images at different scanning positions with the same frame number were rearranged as one data set (e.g. the dataset for 1^st^ frame of the trial at all scanning positions was XYT_1_S_j_ (where X = 184; Y = 128; j = 1, …, 120)). This reconstruction process was repeated for all time points (from 1^st^ to 200^th^ frame) to obtain the entire time course of 3D neural responses.

## Additional Information

**How to cite this article**: Tang, Q. *et al.* *In Vivo* Mesoscopic Voltage-Sensitive Dye Imaging of Brain Activation. *Sci. Rep.* **6**, 25269; doi: 10.1038/srep25269 (2016).
